# Outcomes of surgical treatment for isolated adrenal metastasis from non-small cell lung cancer

**DOI:** 10.3332/ecancer.2021.1322

**Published:** 2021-11-25

**Authors:** Agustin Buero, Walter S Nardi, Domingo J Chimondeguy, Leonardo G Pankl, Gustavo A Lyons, David Gonzalez Arboit, Sergio D Quildrian

**Affiliations:** 1 Thoracic Surgery Department, Buenos Aires British Hospital, Perdriel 74, C1280AEB, Buenos Aires, Argentina; 2 Retroperitoneal, Pelvic and Adrenal Unit, Department of General Surgery, Buenos Aires British Hospital, Perdriel 74, C1280AEB, Buenos Aires, Argentina; 3 Thoracic Surgery Department, Austral University Hospital, Av Juan Domingo Perón 1500, B1629AHJ, Buenos Aires, Argentina; a https://orcid.org/0000-0003-2553-6621

**Keywords:** non-small cell lung cancer, oligometastasis, adrenal metastasis, adrenalectomy

## Abstract

**Objective:**

Long-term survival of patients who undergo surgical resection of isolated adrenal metastasis instead of nonsurgical treatment has shown higher values than those described for stage IVA. The primary endpoint was to evaluate overall survival (OS) of patients with single adrenal metastasis from non-small cell lung cancer (NSCLC), who underwent surgical treatment. The secondary endpoint was to evaluate and compare the OS and disease-free survival (DFS) according to: pathological lung tumour size, histology, lymph node involvement, type of metastasis at the time of diagnosis and laterality of the metastasis according to the primary lung tumour.

**Methods:**

From August 2007 to March 2020, 13 patients with isolated adrenal gland metastasis were identified. We performed a descriptive observational study including patients with diagnosed single adrenal gland metastasis of resectable primary lung cancer and no history of other malignant disease. Clinical data obtained included patient demographics, metastases characteristics, laterality of the metastasis, time between surgeries, length of follow-up, survival status, pathological lung tumour size, histology and lymph node involvement. The variables analysed were OS and DFS.

**Results:**

Median global OS was 31.9 months (interquartile range (IQR), 19.1–51.4). The 2- and 5-year OS estimated was 54% (95% CI: 29.5%–77.4%) and 36% (95% CI: 13.4%–68.1%), respectively. In patients with NSCLC without mediastinal lymph node involvement, we obtain a median OS of 40 months (IQR, 27.4–51.4) and a 2- and 5-year OS estimated of 75% (95% CI: 43.2%–92.2%) and 50% (95% CI: 18.7%–81.2%), respectively. Recurrence was detected in five patients with a median DFS of 11.9 months (IQR, 6–34.2).

**Conclusion:**

The resection of the adrenal metastasis should be considered if the primary lung cancer is resectable. Presence of mediastinal lymph node involvement should be ruled out through invasive staging of the mediastinum before performing adrenal and lung surgery. Proper selection of patients who would benefit from surgery is essential to obtain better survival results.

## Introduction

The adrenal glands are a usual metastatic site of non-small cell lung cancer (NSCLC), even though single extra thoracic metastasis in a single organ is uncommon [1]. Reported incidence of isolated adrenal metastasis from NSCLC is 1.6%–3.5% [2, 3]. Two and five-year overall survival (OS) described by the American Joint Committee on Cancer (AJCC) according to the 8th edition Cancer Staging Manual for NSCLC clinical stage IVA is 23% and 10%, respectively [4]. Although management of NSCLC metastasis is still debated, long-term survival of patients who undergo surgical resection of isolated adrenal metastasis instead of nonsurgical treatment has shown a higher average 2- and 5-year survival than the one described by AJCC. We report our surgical treatment experience for isolated adrenal metastasis from resectable primary lung cancer. The primary endpoint was to evaluate OS of patients with single adrenal metastasis from NSCLC, who underwent surgical treatment. The secondary endpoint was to evaluate and compare the OS and disease-free survival (DFS) according to: pathological lung tumour size (pT), histology, lymph node involvement, metastasis characteristics at the time of diagnosis (synchronous versus metachronous) and laterality of the metastasis according to the primary lung tumour.

## Methods

This is a descriptive observational study performed from August 2007 to March 2020, which included diagnosed isolated adrenal gland metastasis of resectable primary lung cancer and no history of other malignant disease. Patients with mediastinal lymph node involvement in preoperative mediastinal staging were excluded. Data were collected retrospectively from database of two large University Hospitals (Buenos Aires British Hospital and Austral University Hospital). Patients with both synchronous and metachronous metastases were selected. For synchronous, the adrenal gland was initially approached followed by resective lung surgery in all cases. We defined synchronous metastases to those detected within 6 months after resection of the primary tumour, and metachronous metastases when detected 6 months after treatment of the lung primary tumour.

Preoperative staging was performed according to the current International Association for the Study of Lung Cancer (IASLC) guideline at the time of diagnosis [4]. Likewise, all patients were restaged using the 8th edition of TNM [4]. As a requirement for clinical staging, all patients had chest CT, brain magnetic resonance imaging and PET-CT. Invasive mediastinal staging was not performed to all patients. When done, it was through cervical mediastinoscopy or endobronchial ultrasound (EBUS). Patients were suitable for lung surgery when: optimal pulmonary function tests, Eastern Cooperative Oncology Group performance status score 0 and 1 and no cardiological contraindication. All patients had a laparoscopic adrenalectomy prior to the lung cancer approach. An axillar thoracotomy or video-assisted thoracoscopic surgery (VATS) was performed for lung resection with systematic lymph node dissection. Lobectomy was the resection procedure done in all patients. All patients underwent adjuvant chemotherapy.

Clinical data obtained from medical records included patient demographics, type of metastasis (synchronous versus metachronous), laterality of the metastasis, time between surgeries, length of follow-up, survival status, pathological lung tumour size (pT), histology and lymph node involvement. The variables analysed were OS and DFS. OS was defined as the interval from the diagnosis of lung cancer to the last follow-up or death. DFS was considered as the interval between adrenalectomy and the imaging identification of a tumour relapse confirmed through a biopsy.

Statistical analysis was performed using SPSS 13.0 statistical software (SPSS inc., Chicago, IL, USA). All data are presented as median; mean ± standard deviation or counts with percentages. Survival analysis was made using the Kaplan–Meier method and Log-Rank Test.

## Results

Between 2007 and 2020, 13 patients with isolated adrenal metastases of primary lung cancer were diagnosed. Two patients were excluded due to mediastinal lymph node involvement in the preoperative staging leaving a total of 11 patients selected for the study. Demographic and clinical characteristics of the patients are detailed in Table 1. There were nine male and two female patients, with a mean age of 66.9 ± 10 years. Nine patients had synchronous metastasis and two metachronous metastasis. Mean time between surgeries was 29.3 ± 13 days for synchronous and 529 days for metachronous. Left adrenal gland was removed in four cases and right adrenal gland in seven cases. Complete resection was achieved in all cases. In one patient, conversion to open approach was needed due to focal invasion of the inferior vena cava that required a partial resection. This surgery was classified as complete. We had no intraoperative complications and no post-operative deaths. Mean metastatic adrenal tumour size was 4 ± 2.1 cm. Six patients presented ipsilateral metastases, while five contralateral. Pulmonary lobectomy with lymph node evaluation was performed in all patients (systematic nodal dissection or nodal sampling depending on the characteristics of the metastasis).

Preoperative invasive mediastinal staging was performed to seven patients (63.6%), six through videomediastinoscopy and one through EBUS. The mean nodal stations evaluated per patient were 3.4 ± 0.9. None of them had mediastinal lymph node affection. However, in systematic lymph node dissection during resective surgery, one patient had hilar lymph node involvement (pN1) in the definitive pathological result. Of the remaining four patients who did not undergo invasive mediastinal staging, one patient presented mediastinal lymph node involvement (pN2) in the definitive pathological report (patient 10). Histology studies showed NSCLC in nine patients (eight adenocarcinomas, one large-cell) and large cell neuroendocrine carcinoma (LCNEC) in two patients. As adjuvant chemotherapy, most of the patients received pemetrexed + platinum-based antineoplastic drugs. Two patients had to quit due to toxicity (patients 6 and 11). One patient received Pembrolizumab since he presented programmed cell death protein 1 (PD-1) >80% (patient 10).

Recurrence was detected in five patients with a median DFS of 11.9 months (IQR, 6–34.2). All of them had previous adrenal synchronous metastases. Those who presented a worse DFS were patients with a pT > 2 and/or lymph node involvement (patients 7, 8 and10) with a median DFS of 10.1 months. Places of recurrence were: lung-locoregional- (patient 7), bone (patient 10) and central nervous system (patient 8). The remaining two patients had a median DFS of 34.2 months (pT1 without lymph node involvement) and site of recurrence were: lung-locoregional- (patient 1) and bone (patient 4).

**Figure 1.**
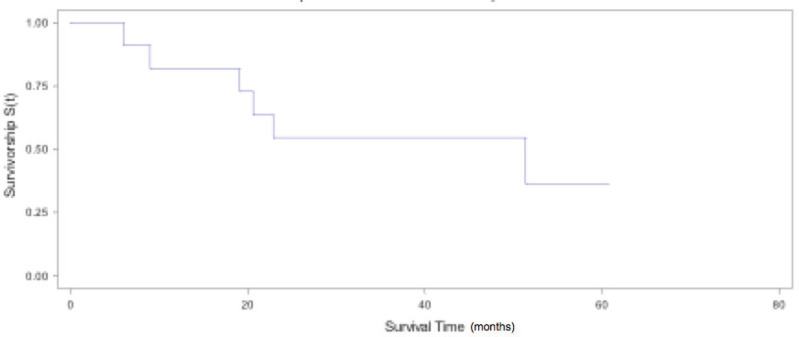
Global OS.

**Figure 2.**
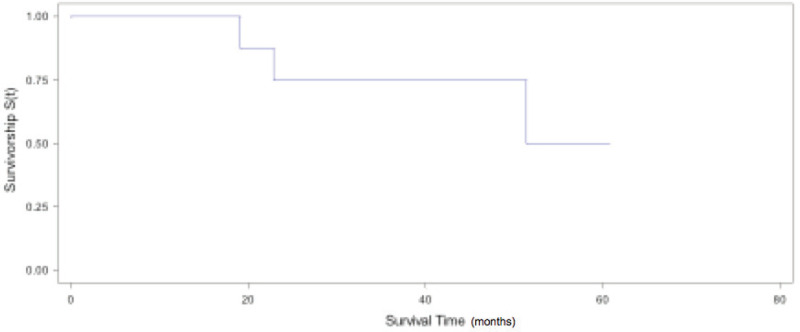
Global OS for NSCLC pN0/1.

Median global OS was 31.9 months (IQR, 19.1–51.4). The 2- and 5-year OS estimated was 54% (95% CI: 29.5%–77.4%) and 36% (95% CI: 13.4%–68.1%), respectively (Figure 1). If we exclude patients with mediastinal lymph node involvement and with LCNEC, we obtain a median OS of 40 months (IQR, 27.4–51.4) and a 2- and 5-year OS estimated of 75% (95% CI: 43.2%–92.2%) and 50% (95% CI: 18.7%–81.2%), respectively (Figure 2). OS and DFS according to pathological lung tumour size (pT), histology, lymph node involvement, metastasis characteristics at the time of diagnosis (synchronous versus metachronous) and laterality to primary are shown in Table 2. Patients with NSCLC, pT1, pN0, metachronic metastases and ipsilateral metastasis had the highest values of DFS and OS.

## Discussion

Oligometastasis was described by Hellman and Weichselbaum [5] as an intermediate state between localised and advanced disease where the number and localisation of metastasis were essential to determine whether they can be treated radically or not. However, until now, the number and location of metastases that a patient must have to be considered as oligometastatic disease are not standardised. In a consensus of experts carried out in 2019, a maximum of five metastases and three organs were proposed, where the lymph node disease was excluded from the definition of oligomestasis but considered as a determining prognostic factor [6]*.* Since the publication by Luketich and Burt [7] in 1996, the idea of including surgery as part of the treatment of isolated adrenal metastases has been consolidated, as long as the primary is resectable. Two recent clinical trials evaluated long-term survival in patients with oligometastasis NSCLC by comparing patients who received only systemic treatment versus systemic treatment plus some local radical therapy [8, 9]. Although the results are within the percentages described by the AJCC for stage IV, the group of patients who received local radical treatment in addition to systematic therapy had a considerably higher survival rate than those who received only systemic treatment. It should be noted that both studies included patients with multiple metastases in different organs. In the latest edition of TNM for lung cancer [4], a new division of extrathoracic metastasis was included, where patients with a single metastatic lesion in a single organ are considered as M1b and multiple metastasic lesions as M1c. Even though management of NSCLC metastases is still debated, there are several publications that describe that adding a radical local treatment such as surgery in stage IVA, more specifically in M1b, improves considerably the OS. For patients with isolated adrenal metastasis who received surgical resection in addition to systemic treatment, an average OS of 27.8% (range 25%–29%) at 5 years is described [10–15]. In our series, we obtained similar percentages, with an estimated global OS at 2 and 5 years of 54% (95% CI: 29.5%–77.4%) and 36% (95% CI: 13.4%–68.1%), respectively. When we excluded patients with LCNEC and with mediastinal lymph node involvement, the percentages were even higher. Although these results are higher than the 2- and 5-year OS described by the AJCC for stage IVA, we do not believe that these results can be extrapolated to other types of single metastases of NSCLC in other organs, mainly the brain where the possibility of performing a resection with margins is limited and where the penetrance of drugs differs from the rest.

Regarding mediastinal staging, there is evidence published supporting those patients with oligometastases without mediastinal lymph node involvement (pN0) have a better long-term prognosis. Therefore, they consider that the absence of mediastinal lymph node involvement is a favourable prognostic factor that would improve OS when compared with those with lymph node involvement. [16, 17]. Although all our patients had non-invasive mediastinal evaluation, only seven patients (63.6%) underwent a preoperative invasive staging to evaluate the mediastinum. Nevertheless, in the histopathological analysis of the hilar and mediastinal nodes of the resective lung surgeries, two patients had lymph node involvement. The patient with pN2 had a DFS and OS of 2 and 6 months, respectively, which is considerably lower than the others. Even though this patient had a negative mediastinal PET-CT, we believe that it is mandatory to perform an invasive method to stage the mediastinum before performing any resection, both lung and metastasis. Preoperative invasive evaluation of the mediastinal lymph nodes is decisive in determining which patients would benefit from a local radical therapy such as surgery. Other prognostic factors associated with improved OS have been identified such as aggressive therapies in the primary lung cancer, female, pT-stage and adenocarcinoma histology [17].

In the meta-analysis published by Tanvetyanon *et al* [13], despite the fact that patients with synchronic metastases had a shorter mean OS than those with metachronous, the 5-year survival estimates were equivalent at 26% and 25%, respectively [13]. In our study, we could not analyse this point since we only had two patients with metachronic metastases. Nevertheless, as shown in Table 2, these two patients had higher OS and DFS than those with synchronous metastases.

It has been held that adrenal metastases present lymphatic dissemination rather than hematogenous spread, mainly those located ipsilateral to the primary lung tumour, having a potential less aggressiveness since it would behave as a locoregional spread and not as a hematogenous metastasis [16, 18]. Our results, in terms of DFS and OS, support this hypothesis since both are higher in patients with ipsilateral metastases (Table 2).

Two large retrospective series of patients with lung cancer oligometastasis were recently published [19, 20]. Although they analyse the role of surgery or radiotherapy in metastases in other sites in addition to the adrenal glands, they describe similar results to those presented in our series. Casiraghi *et al* [19] obtained better prognosis and OS in patients without lymph node involvement and with smaller tumours. Mitchell *et al* [20] conclude that resection of the primary tumour as a component of a comprehensive local consolidative therapy management strategy was associated with long-term OS in a selected subset of patients with NSCLC.

The main limitation of our study is the number of patients. However, if we compare our results with the ones published series of isolated adrenal metastases, we did not find differences in terms of global OS, 2 and 5 years OS and DFS. While there are many retrospective reviews, with known potential biases, we believe that in order to validate our results, further prospective trials are needed since the only one published included only three patients with adrenal metastasis [21].

## Conclusion

Any treatment that increases percentages of OS described by AJCC for patients with clinical stage IVA of NSCLC will be clinically relevant. Resection of the adrenal metastasis should be considered if the primary lung cancer is resectable. Presence of mediastinal lymph node involvement should be ruled out through invasive staging of the mediastinum before performing adrenal and lung surgery. Proper selection of patients who would benefit from surgery is essential to obtain better survival results.

## Conflicts of interest and funding

The authors declare that they have no conflicts of interest or funding.

## Figures and Tables

**Table 1.** Patient’s characteristics.

Patient	Age	Sex	Metastasis	Interval between Surgeries (days)	Histology	pT	pN	DFS (months)	OS (months)	Current status
1	54	M	Synchronic	19	NSCLC	1c	0	38	51.4	Deceased
2	70	M	Synchronic	20	LCNEC	4	0	9.01	9.01	Deceased
3	80	M	Synchronic	35	LCNEC	2a	0	20.6	20.6	Deceased
4	75	M	Synchronic	20	NSCLC	2b	0	30.4	36	Alive
5	60	M	Synchronic	13	NSCLC	1b	0	60.7	60.7	Alive
6	78	M	Synchronic	35	NSCLC	2a	0	44.7	44.7	Alive
7	78	F	Synchronic	34	NSCLC	3	1	10.1	19.1	Deceased
8	65	M	Synchronic	62	NSCLC	3	0	11.9	22.9	Deceased
9	50	F	Metachronic	518	NSCLC	1c	0	51.4	51.4	Alive
10	69	M	Synchronic	26	NSCLC	3	2	2	6	Deceased
11	57	M	Metachronic	540	NSCLC	3	0	31.9	31.9	Alive

DFS: disease-free survival; OS: overall survival; NSCLC: non-small cell lung cancer; LCNEC: large cell neuroendocrine carcinoma

**Table 2.** OS and DFS according to pathological lung tumour size, lymph node involvement, histology, metastasis characteristics and laterality to primary.

	DFS^a^ (months)	OS^a^ (months)
**pT**		
pT1 (3)	50 ± 9	54.5 ± 4.4
pT2 (3)	31.9 ± 9.9	33.7 ± 9.9
pT3 (4)	13.2 ± 9.8	19.2 ± 8.4
pT4 (1)	9.01	9.01
**pN**		
pN0 (9)	32.8 ± 16.5	36.2 ± 16.1
pN1 (1)	10.1	19.1
pN2 (1)	2	6
**Histology**		
NSCLC (9)	30.9 ± 18.8	35.7 ± 16.8
LCNEC (2)	14.8 ± 5.8	14.8 ± 5.8
**Metastasis**		
Synchronic (9)	25.2 ± 18.4	30 ± 18
Metachronic (2)	40.2 ± 11.1	40.2 ± 11.1
**Laterality**		
Ipsilateral (6)	38	43 ± 13.4
Contralateral (5)	13.6 ± 10.4	18.5 ± 10.7

DFS: disease-free survival; OS: overall survival; NSCLC: non-small cell lung cancer; LCNEC: large cell neuroendocrine carcinoma

a Mean ± standard derivation
